# Sub-acute Tamponade and the Value of Point-of-Care Ultrasound for Rapid Diagnosis: A Case Report

**DOI:** 10.5811/cpcem.2017.3.33413

**Published:** 2017-07-14

**Authors:** Daniel C. Kolinsky, Albert J. Kim, Enyo A. Ablordeppey

**Affiliations:** * Washington University School of Medicine, Department of Emergency Medicine, St. Louis, Missouri; † Washington University School of Medicine, Department of Anesthesiology, Division of Critical Care, St. Louis, Missouri

## Abstract

Minoxidil is a strong oral vasodilator that is used to treat patients with hypertension refractory to first-line medications. We report a case of minoxidil-associated subacute cardiac tamponade diagnosed by point-of-care ultrasound (POCUS) in a hypertensive patient. A 30-year-old male with a past medical history of poorly controlled hypertension (treated with minoxidil) and chronic kidney disease presented with 2–3 days of chest pain and shortness of breath with markedly elevated blood pressures. A point-of-care transthoracic echocardiogram revealed a massive pericardial effusion with sonographic tamponade physiology. We review the risk factors for developing pericardial effusions that progress to cardiac tamponade, the utility of diagnosing these patients by POCUS, and the incidence of patients who present with sonographic signs of cardiac tamponade without hypotension.

## INTRODUCTION

In the emergency department (ED), point-of-care ultrasound (POCUS) can help determine the diagnosis, alter the management plan, or assist in procedures. The conventional form ultrasound imaging uses brightness mode (B-Mode, 2D mode), which produces two-dimensional images. However, more recently the utility of motion mode (M-mode) in the ED has been recognized. M-mode is a unique mode of sonographic imaging that displays one-dimensional movement over time. This feature can be used for calculating left ventricular systolic function, looking for pneumothorax, or in this case, diagnosing cardiac tamponade.^1^

Minoxidil is a strong oral vasodilator that acts directly on vascular smooth muscle and is commonly used for treatment of refractory hypertension.^2^ A known potential complication of minoxidil therapy is development of a pericardial effusion, which may progress to cardiac tamponade.^3^–^4^ We report a case of minoxidil-associated cardiac tamponade diagnosed by POCUS in a hypertensive patient.

## CASE REPORT

A 30-year-old male with a past medical history including hypertension, chronic kidney disease (formerly end-stage renal disease on hemodialysis), and congestive heart failure presented with 2–3 days of chest pain associated with dyspnea on exertion and orthopnea. He was on multiple antihypertensive medications including clonidine, lisinopril, labetalol, hydralazine, hydrochlorothiazide, furosemide, and minoxidil.

Upon arrival to the ED, his initial vital signs were temperature of 39.1 degrees Celsius, heart rate of 109 beats per minute, respiratory rate of 38 breaths per minute, blood pressure of 236/155 millimeters of mercury, oxygen saturation of 97% on room air. His physical exam was notable for moderate respiratory distress secondary to tachypnea and accessory muscle use, diffuse expiratory wheezes in all lung fields, and bilateral lower extremity swelling.

**Image 1:**
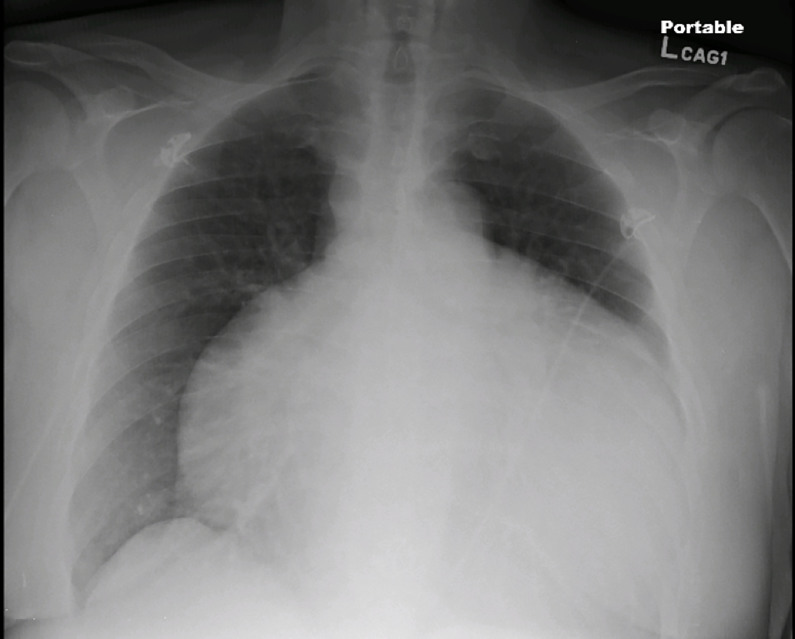
Anterior-posterior chest radiograph demonstrating massively enlarged cardiac silhouette suggestive of a large pericardial effusion

Laboratory evaluation revealed a white blood cell count of 7.4 K/cumm, sodium of 136 mmol/L, and creatinine of 2.64 mg/dL. His troponin was 0.39 ng/ml and brain natriuretic peptide of 233 pg/ml. The electrocardiogram was notable for left ventricular hypertrophy, inferolateral T-wave inversions, and electrical alternans. The patient was started on a nitroglycerin infusion at 60 mcg/min for hypertensive emergency. A chest radiograph (Image 1) revealed an enlarged cardiac silhouette prompting the clinician to perform a point-of-care transthoracic echocardiogram (Images 2B and 3B).

The transthoracic echocardiogram revealed a large pericardial effusion with tamponade physiology, although his blood pressure remained elevated with a peak of 236/155 millimeters of mercury. The nitroglycerin infusion was immediately lowered to 20mcg/min based on the transthoracic echocardiogram findings. The patient was admitted to the cardiac intensive care unit where an urgent pericardiocentesis was performed with approximately one liter fluid removed.

## DISCUSSION

Several case series have been published reporting the association of pericardial effusion with minoxidil.^5^–^7^ The overall incidence of minoxidil-associated pericardial effusion is 3%.^4^ Many patients who take minoxidil for hypertension have co-existing chronic/end-stage renal disease which, in itself, can predispose patients to uremic pericardial effusions.^7^ Thus, it is difficult to estimate the true incidence of pericardial effusions due to minoxidil alone.^7^ In patients undergoing hemodialysis and taking minoxidil compared to patients who are only undergoing hemodialysis, the rate of pericardial effusions was statistically greater (81% vs 23%, p < 0.0005) in those taking minoxidil.^8^

Patients who continue to take minoxidil are at risk of developing a pericardial effusion that can progress to tamponade, especially if they are not followed with surveillance echocardiograms. In the study by Martin et al., of the 52 patients with minoxidil-associated pericardial effusions, 21 of them progressed to pericardial tamponade.^4^ Furthermore, of the 21 patients who developed tamponade, 70% were on hemodialysis compared to 22% who were not.

POCUS is becoming more common in the evaluation of critically ill patients in the ED. Regardless of hemodynamic status, POCUS diagnosis of cardiac tamponade physiology is suggested by three findings: (1) collapse of the right ventricle and right atrium in diastole; (2) exaggerated respiratory variations of transmitral and transtricuspid doppler inflow velocities; and (3) inferior vena cava plethora.^9^ Collapse of the right ventricular free wall in diastole is highly specific, and is the most recognized sonographic sign of cardiac tamponade.^10^ This is demonstrated by placing M-mode over the mitral valve in the parasternal long-axis view (Images 2A and 3A) and observing movement of the right ventricular free wall towards the intra-ventricular septum as the mitral valve opens in early diastole (Image 3B).

Although our patient had a massive pericardial effusion with sonographic signs of cardiac tamponade, his blood pressure was markedly elevated. One retrospective review found that the majority of patients who present with subacute non-traumatic cardiac tamponade will not be hypotensive (15%), but actually normotensive (50%) or even hypertensive (35%).^10^ The existing literature suggests that the incidence of patients with sonographic cardiac tamponade who are hypertensive ranges between 27% to 43%.^12^–^15^ Preexisting hypertension,^14^–^15^ and advanced renal disease,^14^ both of which are present in our patient, are risk factors for developing sonographic cardiac tamponade without hypotension.

CPC-EM Capsule

What do we already know about this clinical entity?

A known potential complication of minoxidil therapy is the development of a pericardial effusion that may progress to cardiac tamponade.

What makes this presentation of disease reportable?

This is the first case of minoxidil-associated subacute cardiac tamponade diagnosed by point-of-care ultrasound in the emergency department.

What is the major learning point?

Point-of-care-ultrasound in motion mode can be used to diagnose sonographic cardiac tamponade in the ED.

How might this improve emergency medicine practice?

Early diagnosis of subacute cardiac tamponade can lead to earlier intervention and avoidance of potential complications, leading to improved outcomes.

We report a case of a patient with minoxidil-associated pericardial effusion with sonographic evidence of tamponade despite being hypertensive, diagnosed by POCUS. Given the increased risk of enlarging pericardial effusion progressing to tamponade with minoxidil use (especially in those with pre-existing renal disease) these patients should have a POCUS performed to screen for sonographic cardiac tamponade even without hypotension.

## Figures and Tables

**Image 2A:**
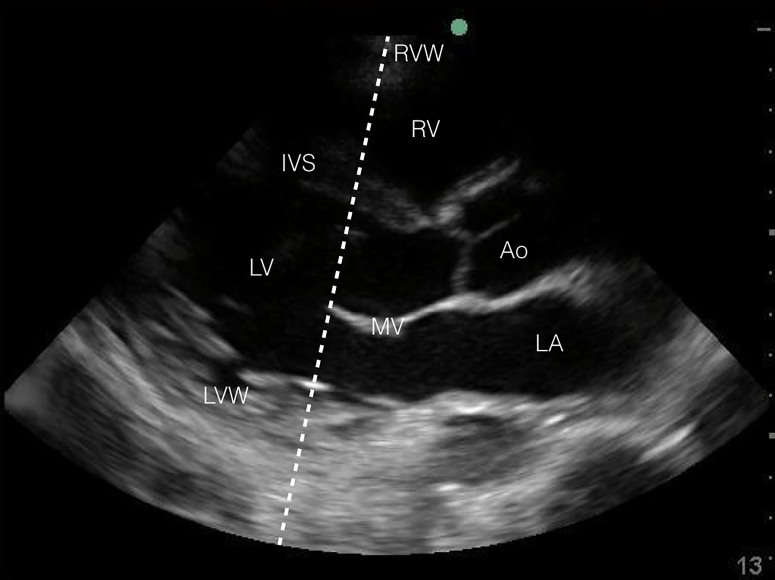
Normal comparison point-of-care transthoracic echocardiogram (parasternal long axis in 2D-mode) – Superimposed (dashed) line overlying the tip of the mitral valve indicating proper motion-mode striker placement. *RVW*, right ventricular wall; *RV*, right ventricle; *IVS*, intraventricular septum; *LV*, left ventricle; *Ao*, aorta; *MV*, mitral valve; *LVW*, left ventricular wall; *LA*, left atrium.

**Image 2B:**
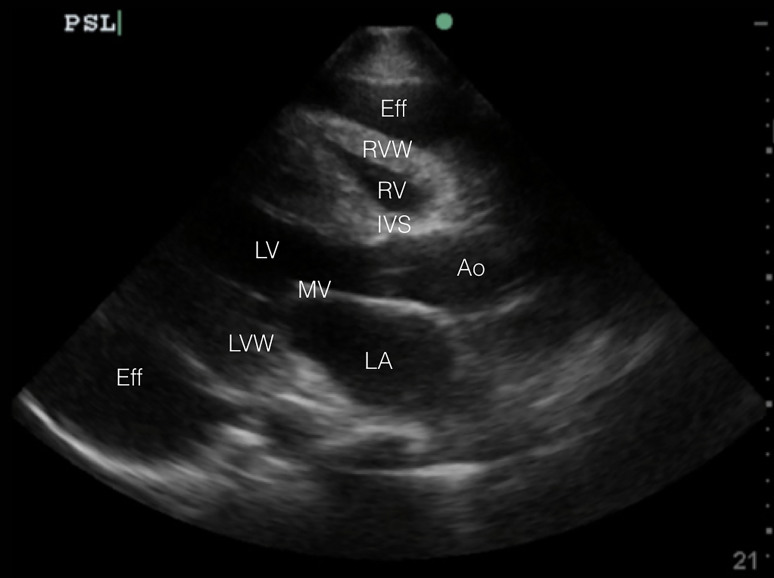
Abnormal point-of-care transthoracic echocardiogram (parasternal long axis in 2D-mode) – circumferential pericardial effusion *Eff*, effusion; *RVW*, right ventricular wall; *RV*, right ventricle; *IVS*, intraventricular septum; *LV*, left ventricle; *Ao*, aorta; *MV*, mitral valve; *LVW*, left ventricular wall; *LA*, left atrium.

**Image 3A:**
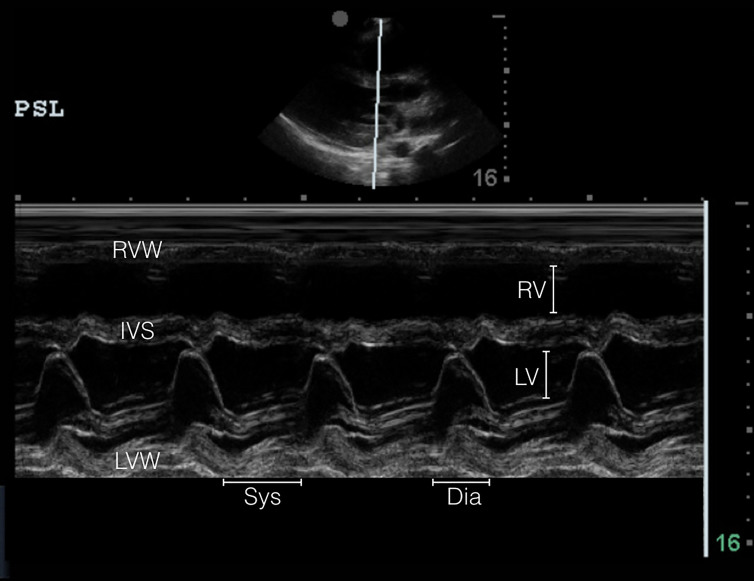
Normal comparison point-of-care transthoracic echocardiogram (parasternal long axis in m-mode) – m-mode tracing showing opening (diastole) or closing (systole) of mitral valve. RV free wall with no diastolic collapse. *RVW*, right ventricular wall; *RV*, right ventricle; *IVS*, intraventricular septum; *LV*, left ventricle; *LVW*, left ventricular wall; *Sys*, systole; *Dia*, diastole.

**Image 3B:**
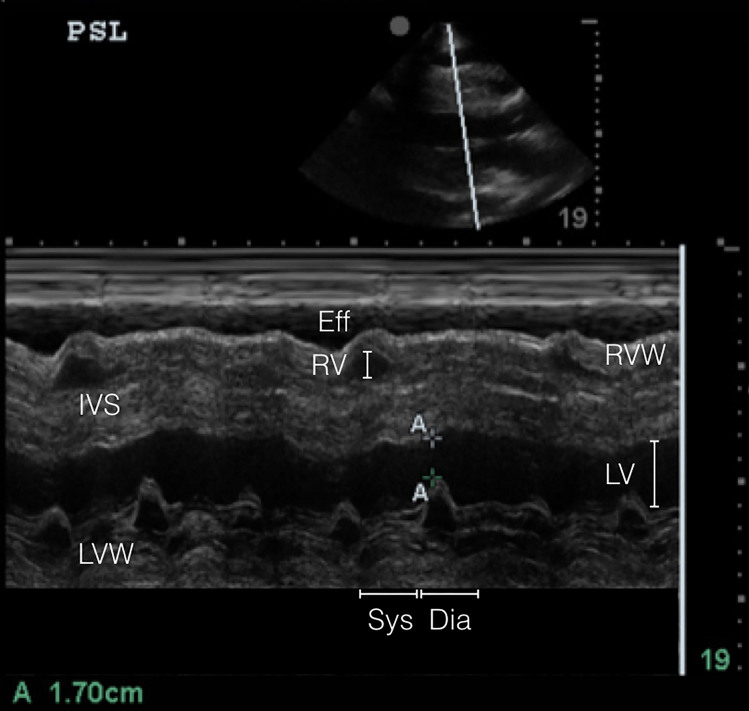
Abnormal point-of-care transthoracic echocardiogram (parasternal long axis in M-mode) - right ventricular wall collapse as the mitral valve begins to open in early diastole. *Eff*, effusion; *RV*, right ventricle; *RVW*, right ventricular wall; *IVS*, intraventricular septum; *LV*, left ventricle; *LVW*, left ventricular wall; *Sys*, systole; *Dia*, diastole.
